# Risk stratification of lateral neck recurrence for patients with pN1a papillary thyroid cancer

**DOI:** 10.1186/s12885-022-10326-8

**Published:** 2022-12-01

**Authors:** Siyuan Xu, Hui Huang, Ying Huang, Xiaolei Wang, Zhengang Xu, Shaoyan Liu, Jie Liu

**Affiliations:** Department of Head and Neck Surgical Oncology, National Cancer Center/National Clinical Research Center for Cancer/Cancer Hospital, Chinese Academy of Medical Sciences and Peking Union Medical College, No. 17, Panjiayuan Nanli, Chaoyang District, Beijing, 100021 People’s Republic of China

**Keywords:** Papillary thyroid carcinoma, Lateral neck recurrence, Central lymph node metastasis, Primary tumor size

## Abstract

**Background:**

Lateral neck is not recommended for dissection in patients with pN1a papillary thyroid cancer (PTC), but its recurrence risk has not been well stratified. We aimed to develop a risk stratification system for lateral neck recurrence in patients with pN1a PTC.

**Methods:**

Patients with pN1a PTC who underwent thyroidectomy and unilateral central compartment dissection from 2000–2016 were enrolled. The association between number of central lymph node metastases (CLNMs) and lateral neck recurrence was comprehensively assessed using a Cox proportional hazards model with restricted cubic spline. Stratification was then performed based on CLNMs and other significant risk factors selected by multivariate analysis. Lateral neck recurrent-free survival (LRFS) rate of each stratification was estimated with Kaplan–Meier curve and comparison was performed using log-rank test.

**Results:**

Ninety-six (3.8%) lateral neck recurrences were identified during a median follow-up of 62 months among a total of 2500 admitted cases. An increasing number of CLNMs was associated with compromised LRFS for up to 6 CLNMs (*P* < 0.001), and CLNMs > 3 indicated significantly worse 5-year LRFS than that of CLNM ≤ 3 (90.6% vs. 98.1%, *P* < 0.001). When stratification with CLNMs and primary tumor size (selected by multivariate analysis, HR (95%CI) = 4.225(2.460–7.256), *P* < 0.001), 5-year LRFS rates of high- (CLNMs > 3 and primary tumor size > 2 cm), intermediate- (CLNMs > 3 and primary tumor size 1–2 cm) and low-risk (primary tumor size ≤ 1 cm or CLNMs ≤ 3) groups were 78.5%, 90.0% and 97.9%, respectively (*P* < 0.05).

**Conclusions:**

The number of CLNMs combined with primary tumor size seems to effectively stratify lateral neck recurrence risk for patients with pN1a PTC.

## Introduction

Papillary thyroid cancer (PTC) is the most common thyroid neoplasm, accounting for 85% of all cases [1, 2]. Cervical lymph node metastasis is common in patients with PTC, but occult metastasis has little effect on the overall prognosis, then neck dissection is only applied when the confirmed nodal disease is detected in the lateral neck [3–5]. However, although this treatment philosophy is reasonable on the whole, some patients have a relatively high risk of lateral neck recurrence, as revealed by some postoperative pathological factors, such as extensive central compartment metastasis, and stratification is necessary to facilitate postoperative management and follow-up.

The overall recurrence risk stratification for PTC has been well established by the 2015 American Thyroid Association (ATA) guidelines, and treatment choices could be made according to the risk groups of the patients [5]. However, the recurrent site and surgical extent are not differentiated in the system, limiting the accurate prediction of lateral neck recurrence. For example, additional lateral neck dissection may compromise regional recurrence risk, especially when a patient has extensive central compartment metastasis, but this factor is not considered for stratification. According to previous studies, central compartment metastasis is regarded as the most important risk factor for lateral neck recurrence [6]. Lim YC et al. found that central lymph node metastasis, especially with extranodal extension, was an independent risk factor for lateral neck recurrence in patients with PTC without clinical evidence of lateral neck metastasis [3]. Moreover, the number of metastatic lymph nodes in the central compartment may be used for risk stratification, which is theoretically reasonable. Previous studies considered the cutoff value of the metastatic number of central compartment lymph nodes as 3, 5, or 6, but their stratification value and relation with other risk factors remain unclear [7–9].

Here, we designed the present retrospective study to comprehensively analyze the relationship between lateral neck recurrence and the number of central compartment lymph node metastases (CLNMs) as well as other risk factors to establish a risk stratification system for lateral neck recurrence in patients with pN1a PTC after thyroidectomy and central compartment dissection.

## Methods and materials

### Patients

This retrospective study was performed on adult patients (18–75 years) who underwent total thyroidectomy or lobectomy and unilateral central neck dissection for pN1a PTC at a single tertiary hospital between January 2000 and December 2016. High-definition cervical ultrasound was routinely performed preoperatively and patients with negative neck were included. Patients with second primary malignancies, poorly differentiated PTC or clinically suspicious cervical lymph nodes on CT/MRI (if performed) were excluded. The study was approved by the Ethics Committee of the Cancer Hospital, Chinese Academy of Medical Sciences. Informed consent was obtained at the time of surgery for general use of clinical information for future studies.

The patient demographics and oncological characteristics were obtained from the institutional database. The primary tumor size, the number of central lymph node dissections (CLNDs), the number of central lymph node metastases (CLNMs), and central lymph node ratio (CLNR, CLNMs/CLNDs) were determined through postoperative pathological examination. The presence of extrathyroidal extension (ETE) and extranodal extension (ENE) of metastatic lymph nodes were examined by microscopic findings. Hashimoto thyroiditis (HT) was determined when the pathological report documented lymphocytic thyroiditis. Decisions on the extent of surgery were at the discretion of the treating physician, with consideration for patient’s preference. Patients were staged according to the American Joint Committee on Cancer staging system (8^th^ edition). Postoperative treatments included conventional thyrotropin suppression at appropriate levels. Radioactive iodine treatment was generally recommended for patients with T4 disease or regional metastasis with a heavy burden during the study period. Physical examination, neck ultrasound and chest CT or radiographs were performed biannually in the first five years and annually after 5 years. The primary endpoint of the study was structural recurrence of the lateral neck, which was determined by either cytological or pathological examination. Lateral neck recurrence-free survival (LRFS) was calculated from the time of surgery for PTC to structural recurrence in the lateral neck.

### Statistical analysis

The association between the number of CLNMs and lateral neck recurrence was assessed using a Cox proportional hazards model with restricted cubic spline (RCS). We then performed stratification analysis corresponding to change points of CLNMs in the relative risk of lateral neck recurrence. Univariable and multivariable Cox proportional hazards models were used to analyze the relationship between clinicopathological variables and LRFS. Variables with *P* < 0.10 in the univariable analyses were selected for the multivariable analyses. The significant risk factors were selected to establish a risk stratification system for lateral neck recurrence. Kaplan–Meier curves were generated to compare the risk of lateral neck recurrence between different risk groups. All statistical analyses were conducted with the R package, version 3.6.2 (R Foundation for Statistical Computing, Vienna, Austria). *P* < 0.05 was considered statistically significant.

## Results

### Patient characteristics

Between 2000 and 2016, a total of 2500 patients were enrolled based on the selection criteria. The cohort’s median age was 41 years (range of 18–75 years), and 1770 (70.8%) were female. The mean primary tumor size was 1.17 ± 0.84 cm, and 50.4% of patients had a tumor less than 1 cm. The mean number of CLNDs, mean number of CLNMs, and mean CLNR were 6.4 ± 4.2, 2.6 ± 2.1 and 0.5 ± 0.3, respectively. Values of CLNDs > 5, CLNMs > 5 and CLNR > 0.5 were achieved in 1234 (49.4%), 237 (9.5%) and 967 (38.7%) patients, respectively. T1, T2, T3 and T4 primary diseases were observed in 2099 (84.0%), 145 (5.8%), 169 (6.8%) and 87 (3.5%) patients, respectively (Table 1). A median follow-up of 62 months showed 136 (5.4%) structural recurrences. More than seventy percent of recurrences (96, 70.6%) occurred in the lateral neck, all of which were ipsilateral to the primary tumor. The LRFS rates at 5 and 10 years of the whole cohort were 96.3% and 89.2%, respectively.

**Table 1:** Study cohort characteristics

Characteristic	N (%)
Total	2500
Age	
< 55 y	2204(88.2)
≥ 55 y	296(11.8)
Sex	
Male	730(29.2)
Female	1770(70.8)
Extent of surgery	
Lobectomy	1827(73.1)
Total thyroidectomy	673(26.9)
Primary tumor size	
≤ 2 cm	2259(90.4)
2–4 cm	211(8.4)
> 4 cm	30(1.2)
Multifocality	794(31.8)
Bilaterality	355(14.2)
Hashimoto thyroiditis	487(19.5)
Extrathyroidal extension	1315(52.6)
Extranodal extension	282(11.3)
Numbers of central lymph node dissections, mean ± SD	6.4 ± 4.2
Numbers of central lymph node metastases, mean ± SD	2.6 ± 2.1
Central lymph node ratio, mean ± SD	0.5 ± 0.3
pT stage	
T1	2099(84.0)
T2	145(5.8)
T3	169(6.8)
T4	87(3.5)
TNM stage	
I	2204(88.2)
II	270(10.8)
III	26(1.0)
Radioactive iodine treatment	275(11.0)

### Relationship between number of CLNMs and LRFS

**Fig. 1:**
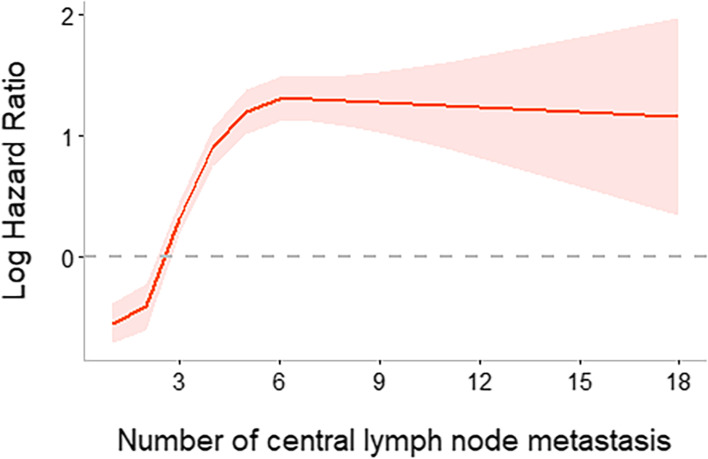
HR and 95% CI (red area) with an increasing number of CLNMs (5 knots were placed)

We used RCS to create a flexible model and visualized the relationship between the number of CLNMs and lateral neck recurrence based on the Cox proportional model. Five knots were placed for RCS, and HR is shown in Fig. 1. There was a significant overall association between the number of CLNMs and LRFS. A significant nonlinear association was also observed (*P* value for the number of CLNMs < 0.001, P for nonlinear < 0.001). This underlines the existence of a change point, which was 6 CLNMs. An increased number of CLNMs was associated with an increased risk of lateral neck recurrence to six CLNMs, after which any additional CLNMs did not confer increased risk.

**Fig. 2:**
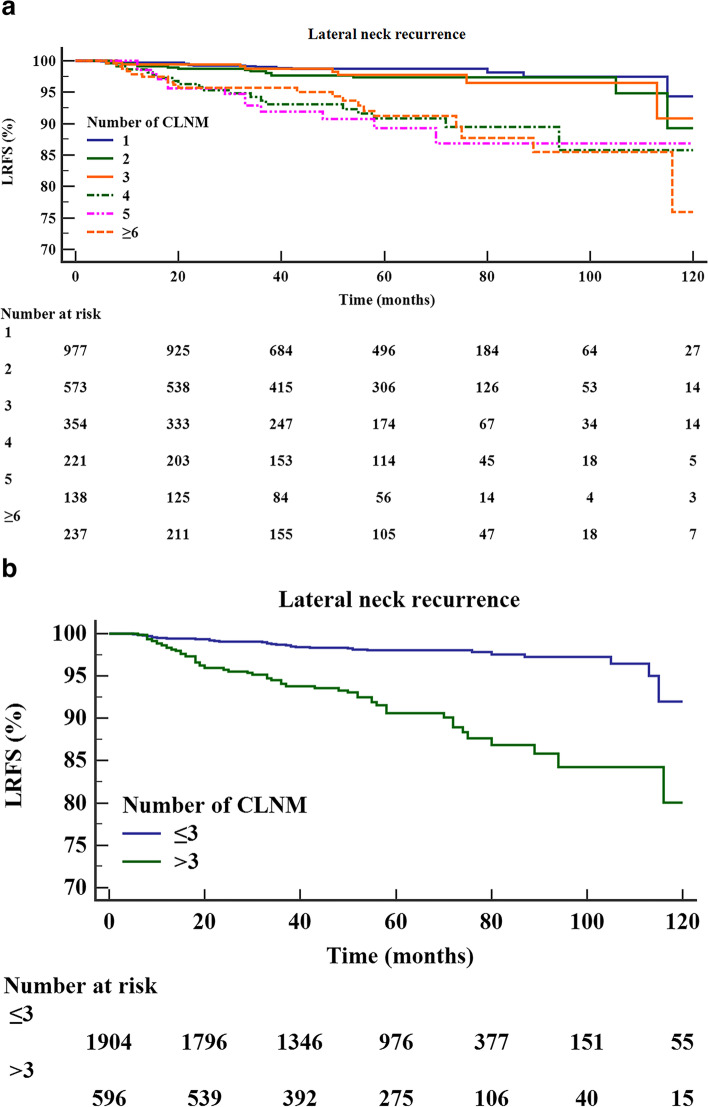
Kaplan–Meier curves for LRFS in patients with different numbers of CLNMs (**A**) and different subgroups of CLNMs (CLNMs ≤ 3 and > 3) (**B**)

According to the nonlinear association between the number of CLNMs and lateral neck recurrence, patients with CLNMs ≥ 6 were classified into one group. They had a similar risk when stratified by different numbers of CLNMs. The 5-year LRFS rates in patients with 1, 2, 3, 4, 5 and ≥ 6 CLNMs were 98.6%, 97.3%, 97.8%, 90.8%, 89.3%, and 91.2%, respectively (Fig. 2A, Table 2). Patients with 4, 5, and ≥ 6 CLNMs had a significantly poorer LRFS than those with 1, 2, and 3 CLNMs did (all *P* < 0.01). No significant difference existed within the 1, 2 and 3 CLNM groups or the 4, 5 and ≥ 6 CLNM groups (Table 2). When regrouping the number of CLNMs, the 5-year LRFS rate was significantly worse for patients with CLNMs > 3 than it was for patients with CLNMs ≤ 3 (90.6% vs. 98.1%, *P* < 0.0001) (Fig. 2B).

**Table 2:** Log-rank analysis stratified by the number of CLNMs demonstrating the risk of lateral neck recurrence

Numbers of central lymph node metastases	5-year LRFS rate	*P* value					
		1	2	3	4	5	≥ 6
1(*n* = 977)	0.986	—	0.212	0.518	0.000	0.000	0.000
2(*n* = 573)	0.973	0.212	—	0.703	0.000	0.000	0.000
3(*n* = 354)	0.978	0.518	0.703	—	0.001	0.000	0.000
4(*n* = 221)	0.908	0.000	0.000	0.001	—	0.452	0.694
5(*n* = 138)	0.893	0.000	0.000	0.000	0.452	—	0.567
≥ 6(*n* = 237)	0.912	0.000	0.000	0.000	0.694	0.567	—

### Other risk factors for lateral neck recurrence

According to the univariate analysis, primary tumor size, multifocality, ETE, ENE, the number of CLNMs and RAI were entered into the multivariate analysis. Primary tumor size > 2 cm (HR = 4.225, 95% CI (2.460–7.256)), multifocality (HR = 1.607, 95% CI (1.055–2.447)), ETE (HR = 1.637,95% CI (1.054–2.544)) and CLNMs > 3 (HR = 4.389, 95% CI (2.887–6.672)) were significantly associated with compromised LRFS (all *P* < 0.05) (Table 3).

**Table 3:** Cox proportional hazards model for lateral neck recurrence

Characteristics	Univariate analysis		Multivariate analysis	
	HR (95% CI)	*P* -value	HR (95% CI)	*P* -value
Male sex	1.113(0.716–1.731)	0.634		
Age (yrs)				
< 55	1(ref)			
≥ 55	0.946(0.505–1.775)	0.864		
Extent				
Lobectomy	1(ref)			
Total thyroidectomy	1.044(0.656–1.662)	0.857		
Primary tumor size				
≤ 1 cm	1(ref)		1(ref)	
1–2 cm	1.875(1.099–3.197)	0.021	1.598(0.918–2.780)	0.097
> 2 cm	4.921(2.911–8.317)	0.000	4.225(2.460–7.256)	0.000
Bilaterality	1.137(0.645–2.007)	0.657		
Multifocality	1.547(1.026–2.334)	0.037	1.607(1.055–2.447)	0.027
Hashimoto thyroiditis	0.814(0.468–1.416)	0.466		
Extrathyroidal extension	1.887(1.237–2.880)	0.003	1.637(1.054–2.544)	0.028
Extranodal extension	1.753(1.023–3.003)	0.041		
CLNMs > 3	4.860(3.228–7.318)	0.000	4.389(2.887–6.672)	0.000
RAI	2.174(0.950–4.976)	0.066		

### Risk stratification system based on the number of CLNMs and primary tumor size

**Fig. 3:**
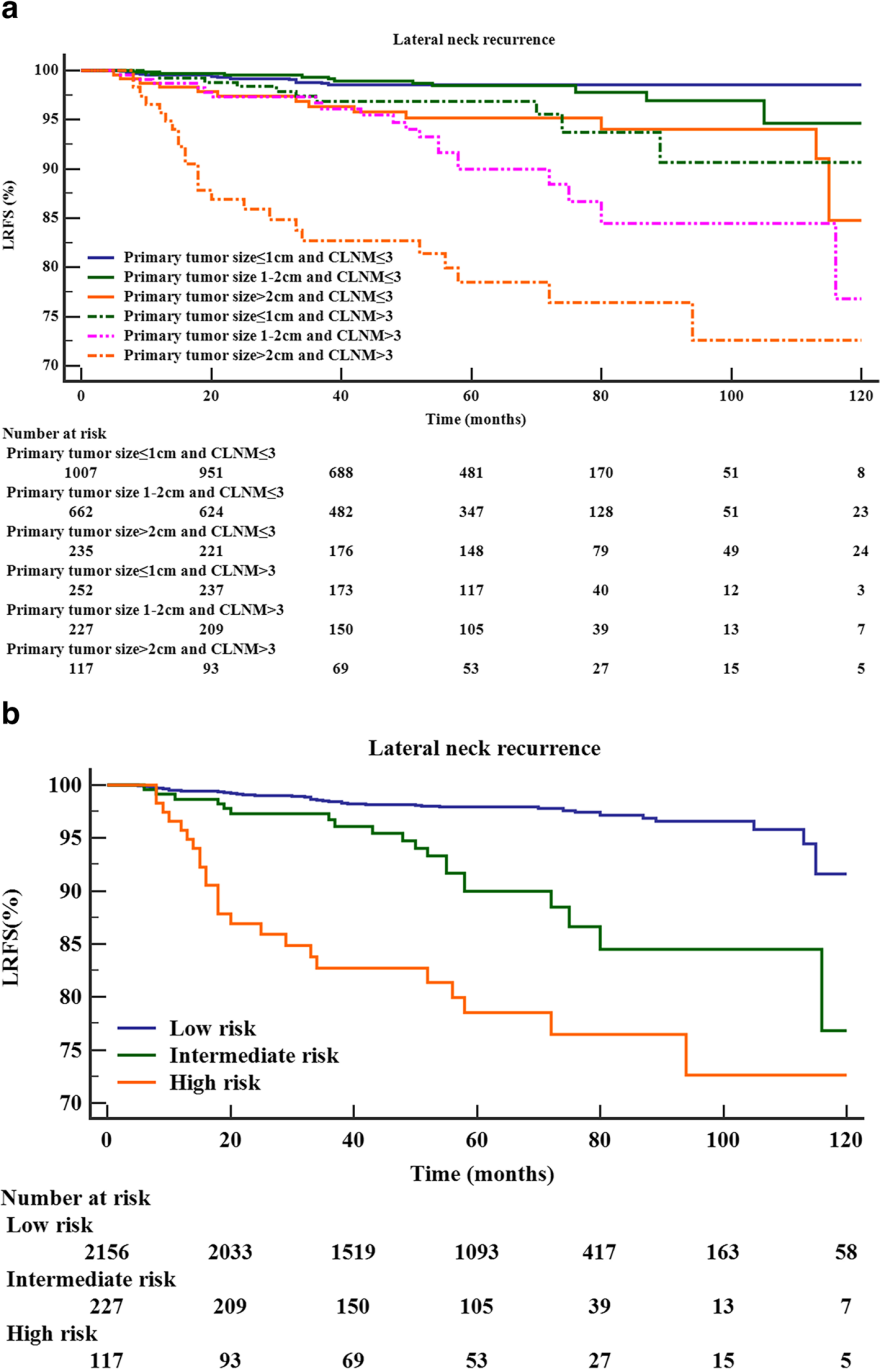
Kaplan–Meier curves for LRFS in patients with different primary tumor sizes and numbers of CLNMs (**A**) and different risk groups based on primary tumor size and the number of CLNMs (**B**)

We selected the number of CLNMs and the other most important risk factor (primary tumor size) to establish a stratification model. Six groups were stratified according to the number of CLNMs (≤ 3, > 3) and primary tumor size (≤ 1 cm, 1–2 cm, > 2 cm). As indicated in Fig. 3A, patients with primary tumor size > 2 cm and CLNMs > 3 showed the poorest LRFS (78.5%), followed by patients with primary tumor size 1–2 cm and CLNMs > 3 (90.0%). Patients with primary tumor size ≤ 1 cm or 1–2 cm and CLNMs ≤ 3 had the best LRFS (98.5% and 98.4%, respectively) (Table 4). To establish the risk stratification system of lateral neck recurrence, patients with primary tumor size > 2 cm and CLNMs > 3, primary tumor size 1–2 cm and CLNMs > 3, primary tumor size ≤ 1 cm or CLNMs ≤ 3 were stratified into high-, intermediate- and low-risk groups with 5-year LRFS rates of 78.5%, 90.0%, 97.9%, respectively (*P* value, low vs. intermediate, < 0.001; low vs. high, < 0.001; intermediate vs. high, 0.005) (Fig. 3B).

**Table 4:** Log-rank analysis stratified by the number of CLNMs and primary tumor size demonstrating the risk of lateral neck recurrence

CLNMs	Primary tumor size	5-year lateral neck RFS	*P* value					
			CLNMs ≤ 3			CLNMs > 3		
			Primary tumor size ≤ 1 cm	Primary tumor size 1-2 cm	Primary tumor size > 2 cm	Primary tumor size ≤ 1 cm	Primary tumor size 1-2 cm	Primary tumor size > 2 cm
≤ 3	≤ 1 cm(*n* = 1007)	98.5%	—	0.657	0.001	0.005	0.000	0.000
	1-2 cm(*n* = 662)	98.4%	0.657	—	0.006	0.022	0.000	0.000
	> 2 cm(*n* = 235)	95.2%	0.001	0.006	—	0.858	0.025	0.000
> 3	≤ 1 cm(*n* = 252)	96.9%	0.005	0.022	0.858	—	0.045	0.000
	1-2 cm(*n* = 227)	90.0%	0.000	0.000	0.025	0.045	—	0.005
	> 2 cm(*n* = 117)	78.5%	0.000	0.000	0.000	0.000	0.005	—

## Discussion

This large cohort study focused mainly on lateral neck recurrence in pN1a PTC patients. Lateral neck recurrence was detected in 3.8% (96/2500) of the patients, which was within the range of 2.4%-4.5% reported by previous studies [3, 8, 10–12] and indicated that overall lateral neck failure was acceptable under the background that prophylactic lateral neck dissection was not performed. However, we also successfully stratified 5% of patients with a high risk of lateral neck recurrence, with a 1/5 to 1/4 possibility of recurrence within 5 years after surgery.

According to the stratification system, some interesting information can be detected. First, although CLNMs > 3 is the most critical risk factor, patients with papillary thyroid microcarcinoma (PTMC) (primary tumor size ≤ 1 cm) still exhibit a low risk for developing lateral neck recurrence (5-year LRFS rate ≥ 95%), which indicates different biological behaviors between PTMC and other PTCs. Moreover, lateral neck recurrence risk increases significantly with increasing primary tumor size in patients with CLNMs > 3. The finding of the predictive value of primary tumor size is helpful to improve the stratification ability with the number of CLNMs alone. Second, ETE was not an important risk factor and was considered essential in the ATA initial risk stratification system. The reason may be that ETE is closely related to the presence and number of CLNMs, which covers the effect of ETE in further predicting lateral neck conditions.

Although several studies have found that the risk of recurrence is associated with the number of CLNDs and CLNR in PTC patients, their effect and optimal cutoff value are still unknown [13]. Barczynski et al. reported that patients with fewer than 6 central lymph nodes had higher recurrence rates in the lateral neck compartment than those with 6 or more central lymph nodes [9]. However, Nam SH et al. found that the lymph node yield was higher in patients with nodal recurrence but did not significantly affect nodal recurrence [14]. Among 467 patients with pN1a, Ryu YJ et al. found that patients with an LNR of more than 0.55 had worse RFS for any lesion and lesion in the lateral neck compartment [12]. A retrospective study involving 2384 consecutive patients who underwent thyroidectomy plus CLND combined with or without lateral LND found that patients with LNR > 0.3 exhibited a 1.7-fold higher risk of posttreatment nodal recurrence (*P* < 0.01) [14]. The variable results among studies may be related to the different inclusion and exclusion criteria, incorporating risk factors and statistical analysis methods. Due to the close relationship among the number of CLNMs, CLND and CLNR, we enrolled the number of CLNMs in the analysis alone, and the prognostic performance and optimal cutoff value of CLNDs and CLNR still need further study.

Currently, patients with papillary thyroid microcarcinoma (≤ 1 cm, PTMC) represent the majority of patients with thyroid cancer. The trend over the years has been for the extent of initial treatment and follow-up to decrease in these patients [4]. When central neck metastasis occurs, active intervention is still under debate. Several studies have shown that microscopic LN metastases have a questionable effect on patient prognosis [4]. In our study, which focused on pN1a PTC patients, an increased number of CLNMs did not significantly increase the risk of lateral neck recurrence in patients with PTMC, and a larger primary tumor size further increased the risk of lateral neck recurrence in patients with CLNMs > 3. Similarly, a retrospective cohort study including 1406 PTC patients who underwent total thyroidectomy and prophylactic bilateral central neck dissection revealed that primary tumor size > 1 cm was significantly associated with lateral neck recurrence (HR = 2.257, 95% CI (1.138–4.476), *P* = 0.020) [12]. These findings supported that active treatment may not be necessary for pN1a PTMC patients, even with a high number of CLNMs, and active surveillance, even prophylactic lateral neck dissection, may be needed for patients with primary tumor size > 2 cm and CLNMs > 3 to improve LRFS.

Nevertheless, our study still has several limitations. First, due to the retrospective nature of these data, some characteristics such as BRAF status and size of CLNMs were not taken into consideration in the study, which may influence the final results. Moreover, it is suggested that superior pole tumors increase the risk of lateral neck lymph node disease due to the proximity of a superior pole tumor to the lateral neck nodes, and tumor location may be a confounding factor with tumor size [15]. Second, due to the conservative surgical strategy during the study period in our institute, only a small proportion of patients with advanced primary disease underwent RAI administration, so RAI turned out to be a risk factor in univariate analysis and not related to lateral neck recurrence in the multivariate analysis. Thus, it is difficult to evaluate the protective value against lateral neck recurrence. Third, other than recurrence, survival outcome is another concern for this topic, but it was not analyzed in our study due to its low incidence rate (0.2%, 5/2500).

Despite these limitations, our study successfully established a simple stratification method, which seems to effectively stratify lateral neck recurrence risk for patients with pN1a PTC.

## Data Availability

The datasets used and/or analyzed during the current study available from the corresponding author on reasonable request.
